# NO_*x*_ degradation ability of S-g-C_3_N_4_/MgAl-CLDH nanocomposite and its potential application in cement-based materials

**DOI:** 10.1039/d3ra04243j

**Published:** 2023-07-18

**Authors:** Zhengxian Yang, Xiaoli Xiong, Xueyuan Yan, Shengyang Luo, Yong Zhang, Bruno Briseghella, Giuseppe Carlo Marano

**Affiliations:** a Joint International Research Laboratory of Deterioration and Control of Costal and Marine Infrastructures and Materials, College of Civil Engineering, Fuzhou University Fuzhou 350108 China y.zhang@fzu.edu.cn; b Department of Structural, Geotechnical and Building Engineering, Politecnico di Torino Corso Duca degli Abruzzi 24-10129 Torino Italy

## Abstract

In this study a new photocatalytic nanocomposite, S-g-C_3_N_4_/MgAl-CLDH, was synthesized and implemented into cement mortar by internal mixing or coating. The photocatalytic NO_*x*_ degradation efficiency of the S-g-C_3_N_4_/MgAl-CLDH and photocatalytic mortar was investigated. The NO_*x*_ degradation efficiency and photoluminescence spectra of S-g-C_3_N_4_/MgAl-CLDH after being immersed in the simulated concrete pore solution were evaluated to assess its chemical stability. The results show that compared with S-g-C_3_N_4_, the S-g-C_3_N_4_/MgAl-CLDH exhibits a narrower bandgap (2.45 eV), a lower photogenerated electron–hole pair recombination rate and a higher specific surface area (36.86 m^2^ g^−1^). After 21 min of visible light irradiation, the NO_*x*_ degradation rate of S-g-C_3_N_4_/MgAl-CLDH achieves 100% as compared to merely 81.5% of S-g-C_3_N_4_. After being submerged in simulated concrete pore solution, the S-g-C_3_N_4_/MgAl-CLDH exhibits only a slight decrease of 5% in degradation rate after 12 min of irradiation, confirming a good compatibility and stability in cement-based materials. The NO_*x*_ degradation ability of the internally mixed mortar is enhanced with an increase in the dosage of S-g-C_3_N_4_/MgAl-CLDH. For coated mortar, in contrast, a decline in NO_*x*_ degradation rate is observed after 5 layers of coating owing to the lower porosity of mortar after excessive coating.

## Abstract 2

Schematic diagram of the synthesis process of S-g-C_3_N_4_/MgAl-CLDH photocatalytic composite.

## Graphical abstract

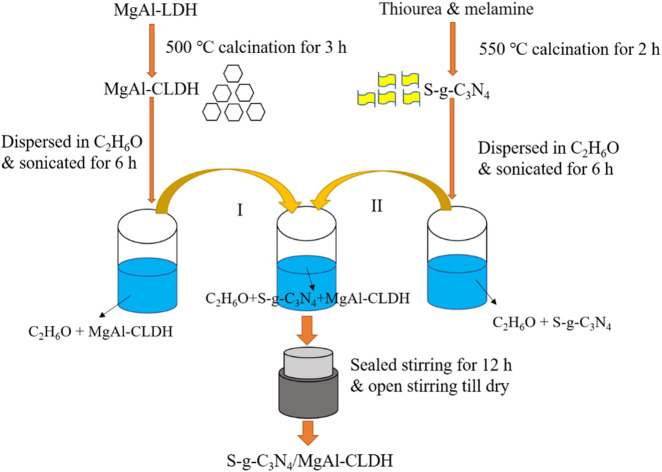

## 1. Introduction

Nitrogen oxides (NO_*x*_) represent a notable category of atmospheric pollutants that give rise to substantial environmental issues, such as the emergence of photochemical smog, the occurrence of acid rain and the development of respiratory diseases in human beings.^1,2^ Adoption of efficacious approaches aiming at preventing or controlling NO_*x*_ pollution is urgently in need. One approach that has gained considerable attention is the implementation of photocatalytic techniques, in particular incorporating photocatalysts into various construction materials such as cement-based materials.^3,4^ Currently, TiO_2_ is known as one of the most widely used photocatalysts. It has however drawbacks such as large band gap width (3.2 eV), high rate of recombination of photogenerated electron–hole pairs, low utilization of visible light and susceptibility to aggregation.^5^ Compared to TiO_2_, a new semiconductor photocatalytic material, graphite carbon nitride (g-C_3_N_4_), has a smaller band gap width (2.7 eV), a wider range of response to visible light and a lower cost.^6^ It is reported that g-C_3_N_4_ can be stimulated by ultraviolet-visible (UV-vis) light and has been utilized in various depollution fields such as degradation of organic pollutants and the mitigation of air pollution.^6^ Nevertheless, due to insufficient absorption of UV-vis light, the g-C_3_N_4_ exhibits a relatively low photocatalytic efficiency, greatly limiting its practical applications.^7^ In this respect, researchers have proposed various modification approaches for g-C_3_N_4_, including non-metal element doping^7^ and semiconductor composites.^8,9^ To begin with, the doping of non-metal elements into g-C_3_N_4_ can reduce the band gap width and broaden the range of light response, thereby enhancing its photocatalytic performance. Liu *et al.*^10^ reported that compared to pure g-C_3_N_4_, Cl-g-C_3_N_4_ exhibited approximately a five-fold increase in specific surface area, leading to enhanced photocatalytic performance. Specifically, the degradation efficiency of Rhodamine B (RhB) using Cl-g-C_3_N_4_ was approximately 12.9 times higher than that of g-C_3_N_4_. Wang *et al.*^11^ synthesized sulfur (S) doped g-C_3_N_4_ (S-g-C_3_N_4_). Compared to g-C_3_N_4_, the S-g-C_3_N_4_ exhibited an absorption edge shift from 455 nm to 470 nm, indicating enhanced visible light absorption capability. Meanwhile the band gap width also reduced from 2.70 eV to 2.63 eV, indicating a decrease in the recombination rate of photogenerated electron–hole pairs. Notably, the photocatalytic efficiency of S-g-C_3_N_4_ in the degradation of methanol (CH_3_OH) was nearly twice that of g-C_3_N_4_.

Another modification method is semiconductor composition, which involves combining two different semiconductor materials with distinct band structures. The energy level difference between the two semiconductors allows for the injection of photogenerated charge carriers from one semiconductor to the energy level of the other, facilitating efficient charge separation and promoting enhanced photocatalytic reaction efficiency.^12^ Gu *et al.*^13^ prepared O-g-C_3_N_4_/TiO_2_ heterostructured photocatalysts using an *in situ* solvothermal method. This composite combines the advantages of both components, such as the higher light utilization efficiency of O-g-C_3_N_4_ and the larger surface area of TiO_2_, effectively enhancing the photocatalytic performance. The results indicate that the optimized O-g-C_3_N_4_/TiO_2_ composite exhibits a photocatalytic performance 6.1 times higher than that of a physical mixture of TiO_2_ and O-g-C_3_N_4_. Xiong *et al.*^14^ synthesized g-C_3_N_4_/CoAl-LDH using the hydrothermal method, and found that the incorporation of g-C_3_N_4_ into CoAl-LDH enhances the photocatalytic activity towards Cr(vi). The enhanced photocatalytic activity is mainly attributed to the synergistic effect of the heterojunction electric field at the interface between g-C_3_N_4_ and CoAl-LDH, which accelerates electron transfer and the separation of photogenerated electron–hole pairs. Zheng *et al.*^15^ prepared MgAl-LDO@g-C_3_N_4_ composite material using a hydrothermal and calcination method and found that MgAl-LDO@g-C_3_N_4_ exhibits superior photocatalytic RhB removal capability compared to Mg-Al-LDO. This can be attributed to the narrow bandgap structure for effective visible light response and the intimate interface for rapid charge transfer and migration.

Noteworthy, the incorporation of g-C_3_N_4_ in cement-based materials suffers from challenges owing to its chemical instability in highly alkaline environment.^16,17^ Great opportunities lie in the utilizations of layered double hydroxides (LDH), a group of compounds consisting of laminates made up of metal oxides and possessing high alkaline resistance. Of particular interest is that the LDH phases have a molecular structure that resembles cement hydration products.^18,19^ As a result, it demonstrates excellent compatibility with cement-based materials. As such, LDH can serve as ideal supportive substrates for photocatalysts.^20,21^ Huang *et al.*^22^ prepared a group of g-C_3_N_4_/CoAl-LDH nanocomposites with different g-C_3_N_4_ content and immersed them into simulated concrete pore solution. They found only a slight increase in photoluminescence (PL) intensity for g-C_3_N_4_/CoAl-LDH after alkaline treatment. In contrast, a significant increase was observed in the case of g-C_3_N_4_. This suggests that the alkaline environment has a lesser impact on the g-C_3_N_4_/CoAl-LDH nanocomposite as opposed to g-C_3_N_4_. Furthermore, the degradation efficiency of NO_*x*_ improves in the g-C_3_N_4_/CoAl-LDH nanocomposites as the g-C_3_N_4_ content increases. This improvement can primarily be attributed to the effective combination of g-C_3_N_4_ with LDH, which facilitates the separation and transfer of photo-induced charge carriers.

The application of g-C_3_N_4_ for the degradation of NO_*x*_ exhibits considerable promise from both environmental and economic perspectives, particularly when implemented in cement-based materials. In order to improve the disadvantages of g-C_3_N_4_ including low visible light utilization, high photogenerated electron–hole pairs recombination rate and poor alkaline resistance, this study employed sulfur for the modification of g-C_3_N_4_ through doping. Then, S-g-C_3_N_4_/MgAl-CLDH nanocomposite was synthesized using electrostatic self-assembly method. A range of analytical techniques was utilized to characterize the physicochemical properties of S-g-C_3_N_4_/MgAl-CLDH, including X-ray diffraction (XRD), X-ray photoelectron spectroscopy (XPS), Fourier transform infrared spectra (FTIR), UV-vis diffuse reflectance spectroscopy (UV-vis DRS), Brunauer–Emmett–Teller (BET), transmission electron microscopy (TEM) and scanning electron microscopy coupled with energy dispersive X-ray (SEM-EDX). Furthermore, the photocatalytic performance of S-g-C_3_N_4_/MgAl-CLDH was analyzed through RhB and NO_*x*_ degradation tests. Lastly, to assess the application potential of S-g-C_3_N_4_/MgAl-CLDH in cement-based materials, the stability of its NO_*x*_ degradation ability in simulated concrete pore solution was examined. The NO_*x*_ degradation ability of cement mortar loaded with S-g-C_3_N_4_/MgAl-CLDH, by internal mixing or coating, was investigated.

## 2. Experimental

### 2.1. Raw materials

All chemical reagents were supported from National Medicine Group Chemical Reagent Co., Ltd., China. The main information of each reagent is shown in Table 1. The ordinary Portland cement (OPC), P.O 42.5, compliant with the Chinese standard GB175-2007, was utilized in the study. The chemical compositions of the OPC were determined using X-ray fluorescence (XRF) analysis and are presented in Table 2. Deionized water and standard sand, as per Chinese standard GB/T 14684-2022, were employed for preparation of cement mortar.

**Table 1:** Information of chemical reagents

	Chemical formula	Purity	Molecular mass
Ethanol anhydrous	C_2_H_6_O	AR	46.07
Thiourea	CH_4_N_2_S	AR	76.12
Calcium hydroxide	Ca(OH)_2_	AR	74.09
Hydrochloric acid	HCl	AR	34.46
Potassium nitrate	KNO_3_	AR	101.10
Rhodamine B	C_28_H_31_CIN_3_O_3_	AR	479.01
Hydrotalcite	Mg_6_Al_2_(OH)_16_CO_3_·4H_2_O	AR	603.98
Melamine	C_3_H_6_N_6_	AR	126.12

**Table 2:** Main chemical composition of OPC (wt%)

CaO	SiO_2_	Al_2_O_3_	Fe_2_O_3_	SO_3_	Other
64.21	20.83	6.22	2.87	1.82	4.05

### 2.2. Synthesis of S-g-C_3_N_4_/MgAl-CLDH composites

For the preparation of g-C_3_N_4_, 2 g of melamine precursor was placed in a muffle furnace, heated up to 550 °C with a heating rate of 3 °C min^−1^, and held for calcination for 2 h. After cooling to room temperature, the light yellow colored g-C_3_N_4_ was obtained, which was then ground into powder. The synthesis of the S-g-C_3_N_4_ involved thermal polymerization, employing melamine as the precursor and thiourea as the source of sulfur. A total of 5 g of melamine precursor and 5 g of thiourea were combined and thoroughly ground to obtain blended mixture. The resulting product was subsequently dried at 60 °C, followed by calcination in a muffle furnace. The calcination process involved heating the mixture at a rate of 3 °C min^−1^ until reaching a temperature of 550 °C, and maintaining this temperature for a duration of 2 h. Subsequently, S-g-C_3_N_4_ with a nanosheet structure was obtained. Similarly, MgAl-CLDH was prepared as follows: 3 g MgAl-LDH was placed in a muffle furnace, heated to 500 °C with a heating rate of 5 °C min^−1^ and held for calcination for 3 h.

**Fig. 1:**
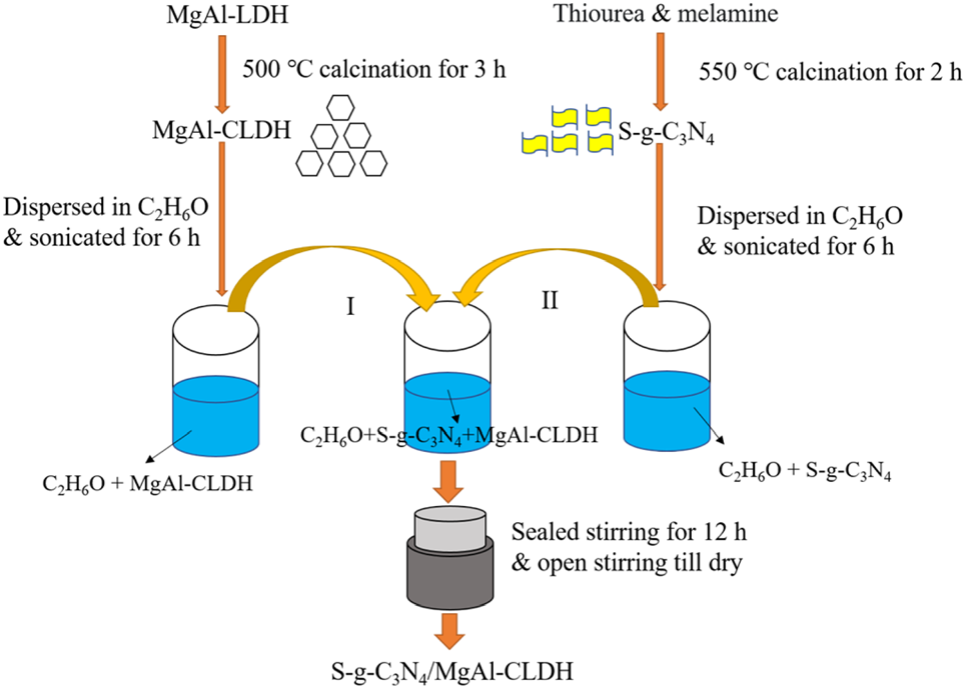
Schematic diagram of the synthesis process of S-g-C_3_N_4_/MgAl-CLDH photocatalytic composite.

The S-g-C_3_N_4_/MgAl-CLDH photocatalytic composites were synthesized *via* electrostatic self-assembly. The preparation procedure, as depicted in Fig. 1, involved the following steps. Firstly, 0.2 g of S-g-C_3_N_4_ was added to a beaker containing 20 mL of anhydrous ethanol. The beaker was then subjected to ultrasonic treatment (with an ultrasonic intensity of 0.96 kW m^−2^) in a water bath for 6 h, resulting in the formation of a well-dispersed S-g-C_3_N_4_ suspension. Similarly, 0.5 g of MgAl-CLDH powder was dispersed in a beaker containing 50 mL of anhydrous ethanol. Subsequently, the suspensions s of S-g-C_3_N_4_ and MgAl-CLDH were combined and placed in an oil bath at 80 °C. The mixture was stirred under sealed conditions for 12 h, followed by open stirring at 80 °C until it was completely dried, resulting in the formation of the desired S-g-C_3_N_4_/MgAl-CLDH photocatalytic composites.

### 2.3. Preparation of photocatalytic cement mortars

Both the internal mixing and coating method were adopted to prepare photocatalytic cement mortars. For the internal mixing method, S-g-C_3_N_4_/MgAl-CLDH (accounting for 0 wt%, 3 wt% and 5 wt% of OPC) was mixed with 135 g of deionized water. The mixture was stirred for 60 min to attain a stable suspension. In the meantime, 450 g of OPC and 1350 g of standard sand were placed into the mixer. Then, dry materials were mixed at a slow speed (140 rpm) for 1 min. Subsequently, the photocatalytic suspension was added into the mixer within 30 s, followed by adding another 135 g of deionized water. Then, the mixture was mixed at a slow speed for 90 s and at a fast speed (285 rpm) for another 90 s. Well-mixed fresh mortar was cast into cube molds with dimensions of 40 mm × 40 mm × 40 mm. After that, the molds were put onto a vibration table for vibrating until bubbles no longer emerge on the mortar surface. During the vibration, a scraper was used to flatten the surface. At one day of curing age, mortar specimens were demolded and transferred into a standard curing room (20 ± 2 °C, >95% RH).

For the coating method, 0.5 g of Ptb emulsion was weighed and dissolved in 100 mL of deionized water and stirred rapidly for 60 min until the emulsion and water got fully mixed to obtain the binding agent. Next, 2 g of S-g-C_3_N_4_/MgAl-CLDH was added to the binding agent and then it was stirred for another 60 min to obtain a uniformly dispersed suspension. Cement mortar with water: cement: sand ratio of 0.6 : 1 : 3 was prepared as per Chinese standard GB/T 17671-1999. Until the age of 28 d, the mortar specimen was coated with the photocatalytic slurry. To make the surface suspension spread evenly, a fine toothpick was used to scrape the surface. The S-g-C_3_N_4_/MgAl-CLDH content of each layer of coating is about 0.02 g. Then, the mortar specimen was dried in an oven with a constant temperature of 60 °C for 10 min. After the coating was completely dry, the next layer of coating was applied, up to a maximum of six layers.

### 2.4. Characterization of physical and chemical properties

Analytical techniques including XRD, FTIR, BET, UV-vis DRS, TEM and SEM-EDX were employed to characterize the photocatalysts. To investigate the crystal plane in each photocatalyst, XRD analysis was conducted using a MiniFlex 600 (Rigaku Co., Ltd., Japan), with a voltage of 40 kV and a current of 40 mA. The diffracted intensity of KαCu radiation was measured across a range of 2*θ* from 5° to 65°, with a scanning rate of 20° min^−1^. By FTIR spectroscopy, the functional groups and chemical bonds of dry photocatalysts were investigated using a Nicolet iS50 (Thermo Fisher Scientific China Co., Ltd.). The scanning range was 400–4000 cm^−1^, with 64 scans and a resolution of 2 cm^−1^. In addition, the heterojunction structure of the sample was observed by TEM using a TECNAI G2 F20 (ThermoFisher, USA). Before TEM analysis, the sample was dispersed in anhydrous ethanol and then deposited on an amorphous carbon grid. The specific surface area and pore size distribution of samples were obtained through BET test using the ASAP 2020 (Micromeritics, USA) automatic specific surface porosity analyzer. Before BET analysis, each sample was heated at 60 °C for 24 h under vacuum. The optical absorbance of dry photocatalysts was examined using a UV-vis spectrometer (Cary 7000, Agilent, USA) within the range of 200–800 nm, and BaSO_4_ was used as the reference. Lastly, the morphology and element mapping of the sample was investigated by SEM-EDX, using a Verios G4 UC (ThermoFisher, USA). Prior to SEM-EDX analysis, powder samples were mounted on double-sided carbon tape and gold-plated for 2 min using a fully automatic sputter coater (Emitech K550X, USA).

### 2.5. Characterization of photocatalytic activity

#### 2.5.1. Photocatalytic degradation of RhB

In a light-proof environment, 0.05 g of S-g-C_3_N_4_/MgAl-CLDH was added to 100 mL of RhB solution with a concentration of 10 mg L^−1^ (*C*_0_). The solution was stirred for 30 min using a magnetic stirrer at a constant temperature of 20 °C until the RhB and photocatalyst in the solution reached the adsorption/desorption equilibrium. At this point, the concentration of RhB was regarded as *C*_q_. Then the beaker with the solution was put under a xenon lamp (300 W), and 5 mL of the solution was taken out every 15 min. The solid and liquid of the reaction solution was separated by a high-speed centrifuge to prevent its further degradation. Finally, the absorbance value of the sample solution at *λ* = 554 nm was tested, and its concentration was determined as *C*_t_. Using eqn (1), the degradation rate *δ* to RhB solution was calculated.1
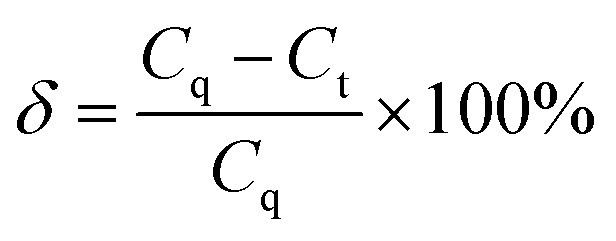


#### 2.5.2. Photocatalytic degradation of NO_x_

**Fig. 2:**
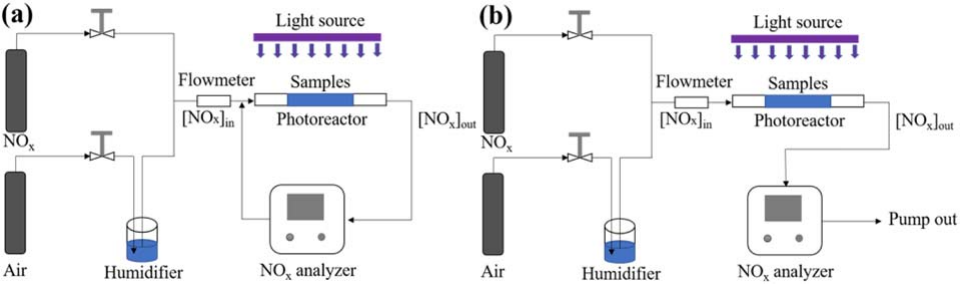
Experimental setup for NO_*x*_ photocatalytic degradation test: (a) cyclic reaction; (b) continuous reaction.

Fig. 2 shows the customized setup for the NO_*x*_ degradation reaction. The setup mainly comprises a NO_*x*_ gas supply, a synthetic air supply, a xenon lamp (300 W), an air humidifier, a gas flowmeter, a reaction chamber and a NO_*x*_ analyzer (Gastiger 6000). NO_*x*_ and air were mixed prior to introduction into the reaction chamber (a sealed quartz glass cylindrical tube). NO_*x*_ analyzer was utilized to monitor real-time NO_*x*_ concentration of the gas out of the chamber. The NO_*x*_ degradation efficiency of the photocatalyst was assessed using the cyclic reaction method (see Fig. 2(a)), whereas the NO_*x*_ degradation efficiency of the photocatalytic mortar was evaluated using the continuous reaction method (see Fig. 2(b)).

In case of cyclic reaction method, 0.5 g of photocatalytic powder was first placed in a quartz groove (40 mm × 20 mm × 10 mm) and then a small amount of anhydrous ethanol was added for ultrasonic dispersion. Next the powder was dried and cooled to room temperature. The prepared sample was put into the reaction chamber. The mixed gas was injected into the chamber, with a gas flow rate of 0.5 L min^−1^. In the meantime, the NO_*x*_ concentration data was recorded every 30 s. When the initial concentration of NO_*x*_ reached a stable equilibrium state, the ventilation was stopped, and the NO_*x*_ concentration value [C_NO_*x*__]_in_ was recorded. To start the photocatalytic oxidation reaction, the xenon lamp was turned on. After illumination for 50 min, the xenon lamp was turn off and the NO_*x*_ concentration value [C_NO_*x*__]_out_ was recorded. Using eqn (2), the degradation rate of the photocatalyst to NO_*x*_ can be calculated.2
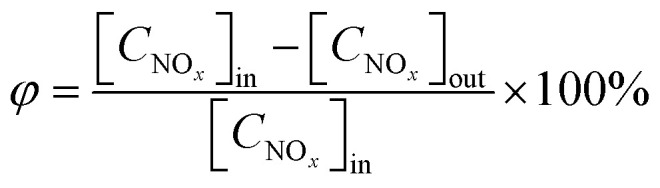
In case of continuous reaction method, the photocatalytic mortar block (40 mm × 20 mm × 5 mm) was placed into the reaction chamber. After the gas reached the preassigned concentration, dark adsorption was performed for 10 min to achieve adsorption balance. Then, the xenon lamp was turned on to activate the photocatalytic NO_*x*_ degradation reaction. The reaction time was 60 min. To regulate the light intensity of the experiment, the distance between the xenon lamp and the reaction vessel was controlled, while the NO_*x*_ gas flow rate was maintained at a constant flow rate of 0.5 L min^−1^. Following the completion of the reaction, the xenon lamp should be deactivated until the NO_*x*_ concentration reverted to its initial instantaneous level prior to the commencement of the reaction. The experimental temperature was regulated at 25 ± 2 °C, while the humidity was maintained at 55 ± 5% RH. The degradation ratio and rate of NO_*x*_ can be calculated as follows:3
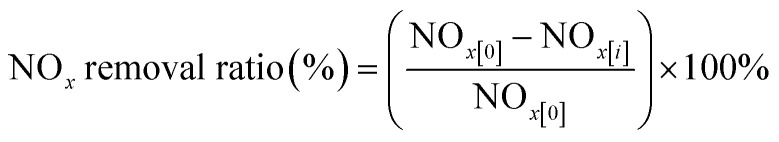
where NO_*x*[0]_ and NO_*x*[*i*]_ represent the initial NO_*x*_ concentration and the NO_*x*_ concentration after the photocatalytic reaction time of *i*, respectively.4
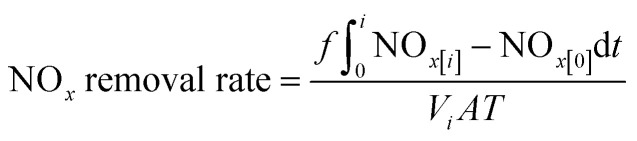
where *f* is the NO_*x*_ gas flow rate under standard conditions, *V*_*i*_ is the volume of 1 mol testing gas under standard conditions (22.4 L), *A* is the surface area of the sample, *T* is the duration of photocatalytic reaction time.

## 3. Results and discussion

### 3.1. Characterization of physicochemical properties of S-g-C_3_N_4_/MgAl-CLDH

#### 3.1.1. XRD analysis

**Fig. 3:**
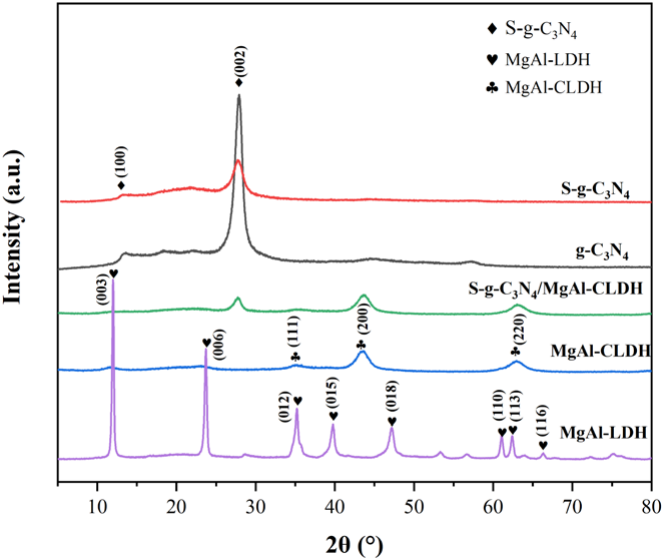
XRD patterns of g-C_3_N_4_, S-g-C_3_N_4_, MgAl-CLDH, MgAl-LDH and S-g-C_3_N_4_/MgAl-CLDH.

Fig. 3 illustrates the XRD patterns of g-C_3_N_4_, S-g-C_3_N_4_, MgAl-CLDH, MgAl-LDH, and S-g-C_3_N_4_/MgAl-CLDH nanocomposites. The characteristic diffraction peaks observed at 2*θ* of 13.0° and 27.3° are attributed to the crystal plane reflections of g-C_3_N_4_, which can be indexed to the (0 0 2) and (1 0 0) planes in JCPDS 87-1526.^23^ Both S-g-C_3_N_4_ and g-C_3_N_4_ exhibit diffraction peaks at the same positions. However, the intensity of the diffraction peaks of S-g-C_3_N_4_ is weakened compared to that of g-C_3_N_4_, indicating a reduction in the crystallinity of g-C_3_N_4_ after sulfur doping. A similar result has been reported in a previous study.^24^ For MgAl-LDH nanosheets, the diffraction peaks observed at 2*θ* of 11.71°, 23.57°, 35.02°, 39.67°, 47.09°, 60.94° and 62.35° represent the crystal plane reflections of the interlayer region containing CO_3_^2−^ hydrotalcite phase, corresponding to the (0 0 3), (0 0 6), (0 1 2), (0 1 5), (0 1 8), (1 1 0) and (1 1 3) planes in JCPDS 035-0964.^25^ Subsequent to the calcination process, the diffraction patterns of MgAl-CLDH reveal peaks at 2*θ* of 36.94°, 42.92° and 62.3°, associated with (1 1 1), (2 0 0) and (2 2 0) planes in JCPDS 87-0653.^26^ These peaks indicate the reflection of crystal planes within the interlayer region, suggesting the absence of the ionic hydrotalcite phase. Furthermore, compared to pure MgAl-LDH, the disappearance of the major characteristic peaks of MgAl-LDH after calcination was observed, resulting in the emergence of distinct characteristic peaks of metal oxides. It is noticeable that S-g-C_3_N_4_/MgAl-CLDH exhibits characteristic diffraction peaks corresponding to both S-g-C_3_N_4_ and MgAl-CLDH, indicating the presence of both phases in the S-g-C_3_N_4_/MgAl-CLDH composite. No additional impurity peaks are observed in the XRD spectra, suggesting the absence of crystalline impurities in the S-g-C_3_N_4_/MgAl-CLDH, thus confirming its successful synthesis and high purity.

#### 3.1.2. FTIR spectra

**Fig. 4:**
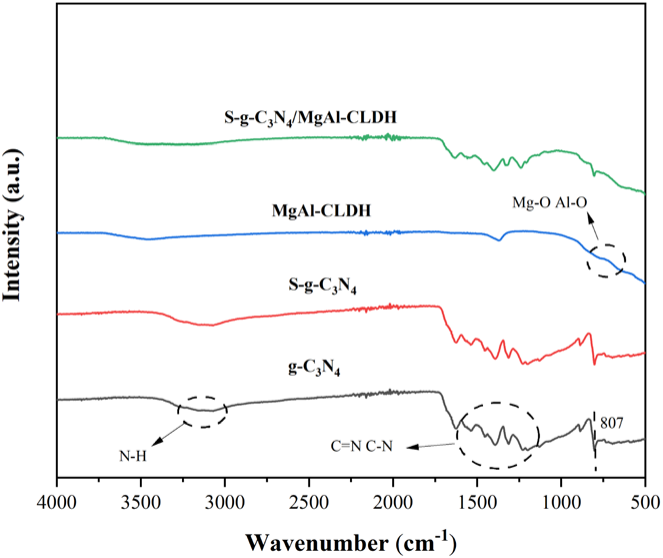
FTIR spectra of g-C_3_N_4_, S-g-C_3_N_4_, MgAl-CLDH and S-g-C_3_N_4_/MgAl-CLDH.

Fig. 4 depicts the FTIR spectra of g-C_3_N_4_, S-g-C_3_N_4_, MgAl-CLDH and S-g-C_3_N_4_/MgAl-CLDH nanocomposites. It can be observed that the peak around 807 cm^−1^ in g-C_3_N_4_ corresponds to the characteristic breathing-vibration mode of the intralayer heterocyclic triazine units, forming a hexagonal structure, and the peaks between 1200 cm^−1^ to 1650 cm^−1^ correspond to the typical stretching vibrations of C=N and the heterocyclic vibrations of C–N.^27^ Furthermore, the spectra of g-C_3_N_4_ and S-g-C_3_N_4_ exhibit a similar profile, indicating that the sulfur doping does not alter the chemical structure of g-C_3_N_4_. The peaks observed at 509 cm^−1^ and 668 cm^−1^ in MgAl-CLDH can be attributed to the stretching vibrations of Al–O, while the peak at 856 cm^−1^ can be attributed to the stretching vibrations of Mg–O.^28^ The characteristic peaks of S-g-C_3_N_4_/MgAl-CLDH are similar to those of S-g-C_3_N_4_. This indicates that MgAl-CLDH is successfully loaded onto the surface of S-g-C_3_N_4_, and the chemical structure of S-g-C_3_N_4_ is not disrupted during the composite formation with MgAl-CLDH. However, the relative peak intensities in the range of 1240 cm^−1^ to 1640 cm^−1^ are much weaker in S-g-C_3_N_4_/MgAl-CLDH than in S-g-C_3_N_4_. This could be attributed to the formation of chemical bonding between the Mg in MgAl-CLDH and the N in S-g-C_3_N_4_.^29^ Furthermore, the FTIR spectra of the S-g-C_3_N_4_/MgAl-CLDH sample exhibit vibrational bands corresponding to both S-g-C_3_N_4_ and MgAl-CLDH, proving the formation of a heterostructure in S-g-C_3_N_4_/MgAl-CLDH.

#### 3.1.3. TEM analysis

**Fig. 5:**
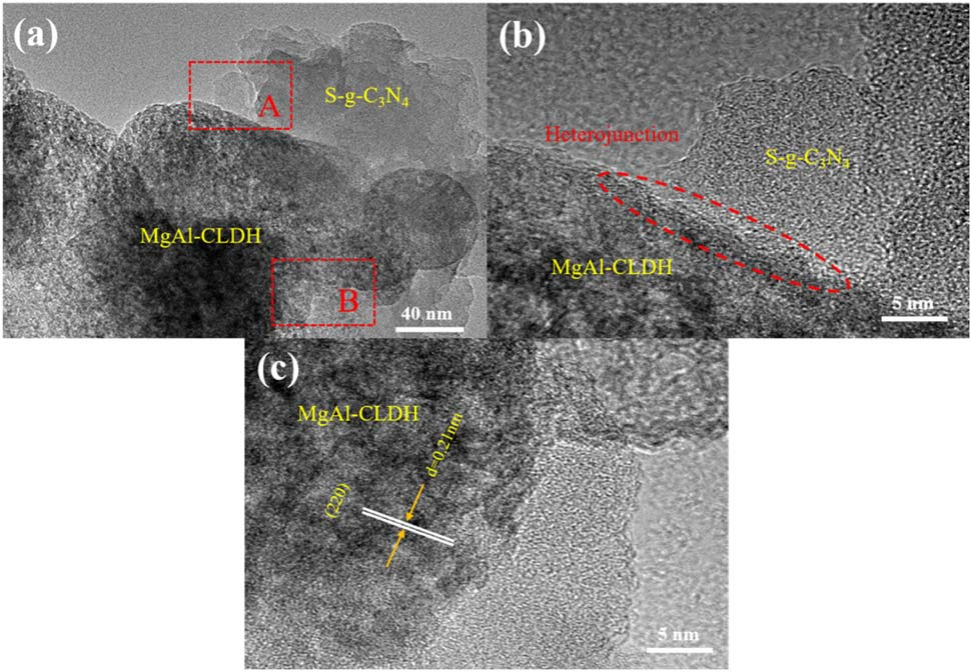
TEM images of S-g-C_3_N_4_/MgAl-CLDH: (a) under low magnification; (b) section A under high magnification; (c) section B under high magnification.

To investigate the morphology and structural characteristics of the S-g-C_3_N_4_/MgAl-CLD, TEM analysis was employed, and the results are shown in Fig. 5. The MgAl-CLDH exhibits a hexagonal morphology with a lateral dimension of approximately 50 nm.^30^ It can be observed that MgAl-LDH undergoes collapse of its structure after calcination, resulting in the formation of irregularly shaped two-dimensional nanosheets of MgAl-CLDH. The surface of S-g-C_3_N_4_ appears relatively smooth, exhibiting a typical amorphous two-dimensional nanosheet structure. By loading MgAl-CLDH onto the S-g-C_3_N_4_, the S-g-C_3_N_4_/MgAl-CLDH heterostructure is obtained (see Fig. 5(b)). As shown in Fig. 5(c), lattice fringes with a lattice spacing of 0.21 nm are observed on the (2 2 0) crystal plane of the Mg(Al)O cubic phase,^31^ while the amorphous domains, due to their low crystallinity, are attributed to S-g-C_3_N_4_, confirming the presence of both S-g-C_3_N_4_ and MgAl-CLDH. According to what have been observed in TEM images, it is safe to say that the MgAl-CLDH nanosheets are successfully loaded onto the surface of S-g-C_3_N_4_ through electrostatic self-assembly, resulting in the formation of a heterostructure, S-g-C_3_N_4_/MgAl-CLDH. This 2D/2D heterostructure with an intact interface facilitates the transfer and separation of photogenerated charge carriers.^32^

#### 3.1.4. BET test

**Fig. 6:**
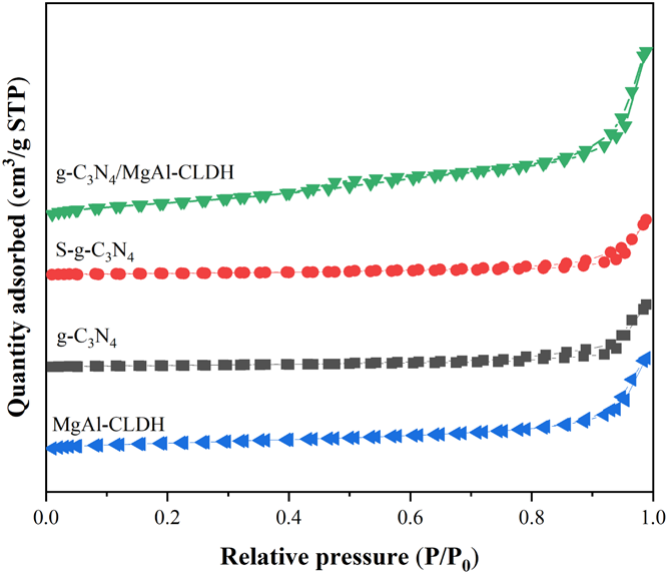
Nitrogen adsorption–desorption isotherms of g-C_3_N_4_, S-g-C_3_N_4_, MgAl-CLDH and S-g-C_3_N_4_/MgAl-CLDH.

**Fig. 7:**
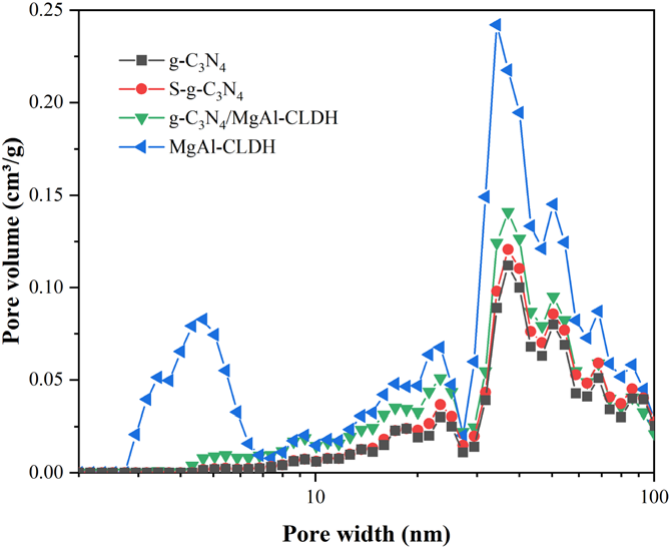
Pore size distribution of g-C_3_N_4_, S-g-C_3_N_4_, MgAl-CLDH and S-g-C_3_N_4_/MgAl-CLDH.

Fig. 6 presents the nitrogen adsorption–desorption isotherms of g-C_3_N_4_, S-g-C_3_N_4_, MgAl-CLDH and S-g-C_3_N_4_/MgAl-CLDH. The slope in the BET equation is used to calculate the amount of monolayer adsorption. In combination with the area occupied by individual molecules on the adsorbent surface, the specific surface area of the sample can be calculated. Meanwhile, the pore size distribution can be calculated according to the BJH method. Table 3 provides the pore diameter, specific surface area and pore volume of each photocatalyst. The specific surface area and pore volume of the S-g-C_3_N_4_ are 12.55 m^2^ g^−1^ and 0.023 cm^3^ g^−1^, respectively. After the electrostatic loading of MgAl-CLDH nanosheets onto the surface of S-g-C_3_N_4_, the specific surface area and pore volume of S-g-C_3_N_4_/MgAl-CLDH are significantly increased, reaching respectively 2.94 times and 3.95 times that of S-g-C_3_N_4_. The incorporation of S-g-C_3_N_4_ leads to the formation of an outward-expanding structure on the surface of MgAl-CLDH, resulting in the enhanced generation of pores and an increased specific surface area of the sample. It has been reported that larger pore volume and higher specific surface area are beneficial for enhancing the absorption of UV-vis light and increasing the number of active catalytic sites.^33^ The pore size distributions are derived from desorption branches of isotherms using BJH method, as shown in Fig. 7. The pore size distribution of S-g-C_3_N_4_ is mainly concentrated between 2 nm and 30 nm. In comparison to S-g-C_3_N_4_, the relative volume of large pores (>50 nm) in S-g-C_3_N_4_/MgAl-CLDH has significantly increased, which can enlarge the exposed surface area and enhance light utilization. Therefore, it can be inferred that the loading of MgAl-CLDH will be beneficial for improving the photocatalytic activity of S-g-C_3_N_4_.

**Table 3:** Pore size, specific surface area and pore volume of different photocatalysts

Samples	*S*_BET_ (m^2^ g^−1^)	Pore volume (cm^3^ g^−1^)	Average pore diameter (nm)
g-C_3_N_4_	7.54	0.014	18.55
S-g-C_3_N_4_	12.55	0.023	17.32
MgAl-CLDH	54.79	0.047	9.68
S-g-C_3_N_4_/MgAl-CLDH	36.86	0.091	13.63

#### 3.1.5. UV-vis DRS analysis

The most critical factors governing the photocatalytic activity can be ascribed to the light absorption and the capacity for transferring excited electrons in semiconductors. The optical characteristics of the samples were determined by measuring their UV-vis DRS. Additionally, the bandgap width of the photocatalytic material was calculated using the Tauc plot function, as shown in eqn (5).^34^

5 *αhν* = *K*(*hν* − *E*_g_)^2/*n*^

where *α* represents the absorption coefficient, *h* represents the Planck constant, *v* represents the frequency of light, *K* represents a proportionality constant, *E*_g_ represents the bandgap width of the photocatalytic material. Additionally, the characteristics of semiconductor electron transitions determine the exponent *n* (g-C_3_N_4_ is a direct bandgap semiconductor, allowing direct electron transitions, hence *n* is 1).

**Fig. 8:**
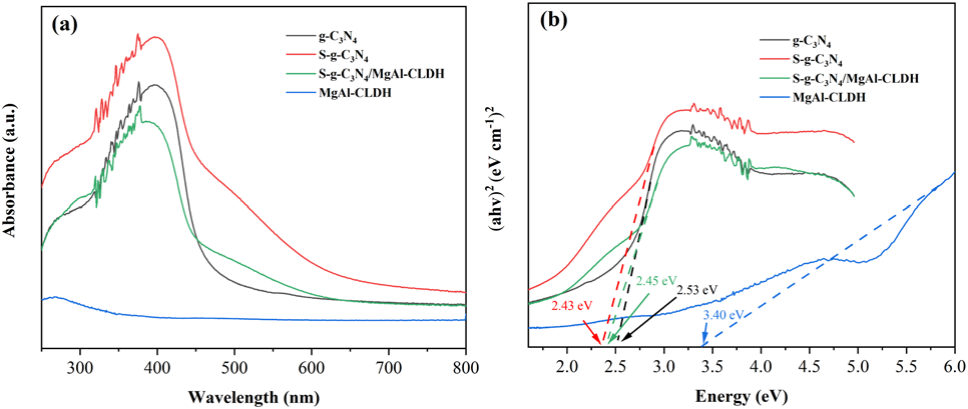
(a) UV-vis absorption spectra and (b) Tauc plot of g-C_3_N_4_, S-g-C_3_N_4_, MgAl-CLDH and S-g-C_3_N_4_/MgAl-CLDH.

Fig. 8(a) and (b) show the UV-vis diffuse reflectance spectra and bandgap width curve of each photocatalyst, respectively. It can be seen that compared to g-C_3_N_4_, S-g-C_3_N_4_ exhibits a redshift in the absorption edge. Specifically, the maximum absorption edge positions of g-C_3_N_4_ and S-g-C_3_N_4_ are 455 nm and 470 nm, respectively. In addition, the widened absorption range and the heightened absorption peak observed in S-g-C_3_N_4_ suggest an increased capacity for light absorption. The bandgap widths of S-g-C_3_N_4_ and g-C_3_N_4_ are determined to be 2.43 eV and 2.53 eV, respectively. This indicates that sulfur doping can reduce the bandgap width of g-C_3_N_4_, thereby effectively separating photogenerated electron–hole pairs and enhancing the light response range. On the other hand, the weak absorption phenomenon of the MgAl-CLDH derived metal oxide under UV light (below 400 nm) indicates its low photocatalytic performance. Compared to MgAl-CLDH and g-C_3_N_4_, the S-g-C_3_N_4_/MgAl-CLDH shows significantly different absorption curves. Firstly, S-g-C_3_N_4_/MgAl-CLDH exhibits a noticeable absorption band near 465 nm within the visible light range, and its bandgap width is calculated to be around 2.45 eV. Secondly, S-g-C_3_N_4_/MgAl-CLDH demonstrates a redshift in the absorption wavelength. As a result, under the same UV-vis light absorption intensity, S-g-C_3_N_4_/MgAl-CLDH exhibits stronger absorption in the visible light range than g-C_3_N_4_ and MgAl-CLDH. Additionally, S-g-C_3_N_4_/MgAl-CLDH exhibits a wider absorption peak in the range of 450 nm to 550 nm. Accordingly, the electrostatic self-assembly of S-g-C_3_N_4_ with MgAl-CLDH effectively improves the material's optical properties and enhances the range of visible light absorption, and meanwhile increases light utilization efficiency and generates more photogenerated electron–hole pairs, thereby significantly enhancing the photocatalytic activity.

#### 3.1.6. SEM-EDX analysis

**Fig. 9:**
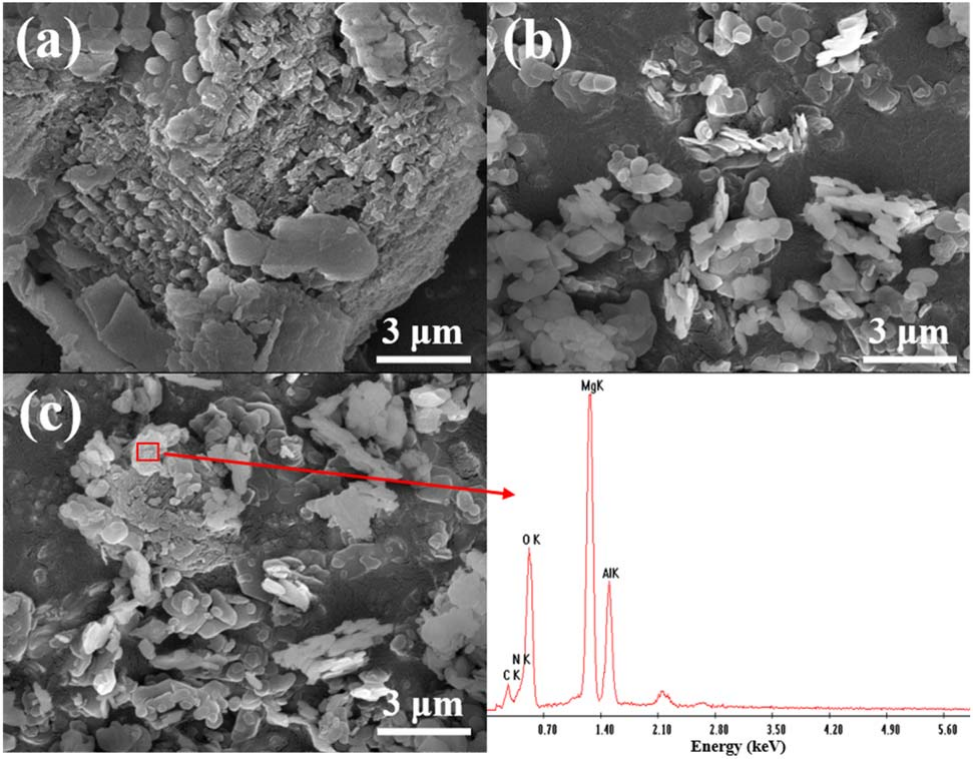
SEM image: (a) S-g-C_3_N_4_; (b) MgAl-CLDH; (c) S-g-C_3_N_4_/MgAl-CLDH.

Fig. 9 shows the SEM images of S-g-C_3_N_4_, MgAl-CLDH and S-g-C_3_N_4_/MgAl-CLDH. As shown in Fig. 9(a), S-g-C_3_N_4_ exhibits an irregular two-dimensional layered structure with relatively thick layers. Additionally, the surface of S-g-C_3_N_4_ appears rough and porous, primarily due to the generation of a significant amount of carbon dioxide and ammonia during the preparation process, which promotes the formation of porous structures. Meanwhile, the MgAl-CLDH monomer consists of hexagonal shapes with relatively uniform sizes, approximately in several micrometers (see Fig. 9(b)). After the electrostatic self-assembly of S-g-C_3_N_4_ and MgAl-CLDH, it can be observed that the surface of S-g-C_3_N_4_ is loaded with a large number of MgAl-CLDH layered structures (see Fig. 9(c)). Compared to the monomer structure of S-g-C_3_N_4_, S-g-C_3_N_4_/MgAl-CLDH nanocomposite exhibits a significantly larger particle size, enhanced porosity and irregular morphology, promoting a face-to-face contact between the 2D materials. This facilitates an improved charge transfer rate of the photocatalyst^35^ and provides more active sites and larger specific surface area for photocatalytic reactions.

### 3.2. Characterization of photocatalytic activity of S-g-C_3_N_4_/MgAl-CLDH

#### 3.2.1. Degradation effect of S-g-C_3_N_4_/MgAl-CLDH on RhB

**Fig. 10:**
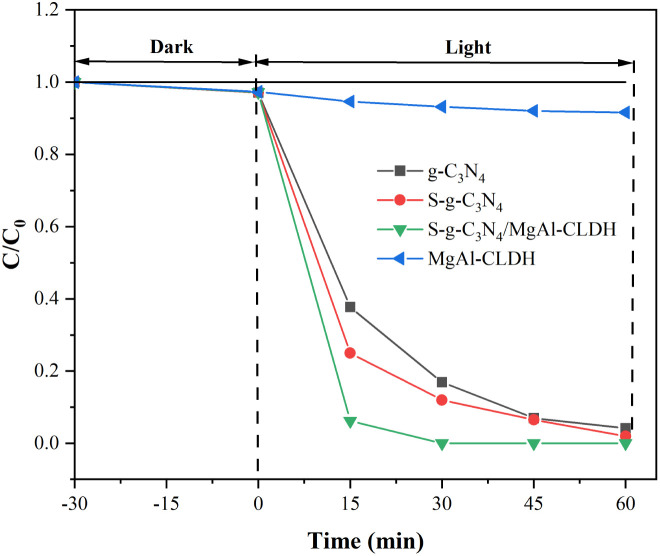
Photocatalytic degradation of RhB by g-C_3_N_4_, S-g-C_3_N_4_, MgAl-CLDH and S-g-C_3_N_4_/MgAl-CLDH.

To characterize the photocatalytic activity of different photocatalytic materials, RhB was selected as one of the pollutants, and its degradation effect is shown in Fig. 10. As can be seen, under the dark environment, the concentrations of RhB in different solution were all reduced, mainly owing to the physical adsorption on the surface of the photocatalytic materials. The g-C_3_N_4_ exhibited the lowest RhB degradation rate due to its limited light energy absorption capacity. To be specific, after 30 min of irradiation, the RhB degradation rate of g-C_3_N_4_ reached 83.1% only. However, the RhB degradation rate of S-g-C_3_N_4_ attained 88%, indicating modification with sulfur has positively contributed to the photocatalytic activity of g-C_3_N_4_. Similar results have been reported by Hu *et al.*^36^ It is noteworthy that the S-g-C_3_N_4_/MgAl-CLDH nanocomposite exhibits remarkable degradation ability towards RhB, particularly at an early stage. The degradation rate of S-g-C_3_N_4_/MgAl-CLDH at 15 min was 15.67 times that of MgAl-CLDH and 1.25 times that of S-g-C_3_N_4_. Complete degradation of RhB in the solution is achieved within 30 min. The exceptional performance can be attributed to the interfacial effect between S-g-C_3_N_4_ and MgAl-CLDH, which facilitates electron separation and transfer, thus significantly enhancing the photocatalytic activity. Even after 60 min of irradiation, there still remains some residual RhB in the solution regardless of S-g-C_3_N_4_ or MgAl-CLDH as the photocatalyst.

#### 3.2.2. Degradation effect of S-g-C_3_N_4_/MgAl-CLDH on NO_*x*_

**Fig. 11:**
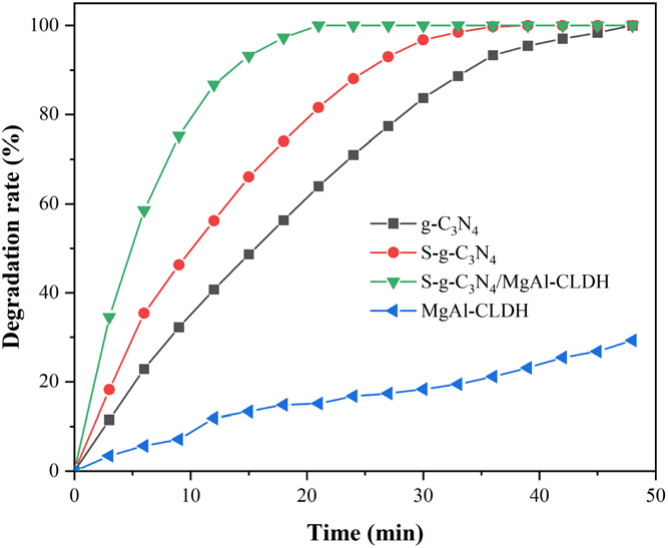
Photocatalytic degradation of NO_*x*_ by g-C_3_N_4_, S-g-C_3_N_4_, MgAl-CLDH and S-g-C_3_N_4_/MgAl-CLDH.

The photocatalytic activities of each photocatalyst were also investigated in terms of NO_*x*_ degradation, and the result is shown in Fig. 11. Among the tested photocatalysts, MgAl-CLDH exhibits the least photocatalytic activity, akin to the findings observed for RhB degradation. Due to the high photogenerated electron–hole pair recombination rate of g-C_3_N_4_ and its low absorption of visible light, the g-C_3_N_4_ shows the second lowest photocatalytic performance. Conversely, the combining of sulfur brings about a specific positive influence on the photocatalytic activity of g-C_3_N_4_. After 21 min of exposure, the photocatalytic degradation rates of g-C_3_N_4_ and S-g-C_3_N_4_ reached 64.0% and 81.5%, respectively. By combining S-g-C_3_N_4_ and MgAl-CLDH, the photocatalyst (S-g-C_3_N_4_/MgAl-CLDH) with exceptional NO_*x*_ degradation ability is obtained. Specifically, the NO_*x*_ degradation rate could reach 100% at 21 min for S-g-C_3_N_4_/MgAl-CLDH. The enhanced photocatalytic performance of S-g-C_3_N_4_/MgAl-CLDH is achieved through the reduction of the photogenerated electron–hole pair recombination rate. This reduction accelerates the photocatalytic redox reaction, resulting in an overall improvement in the photocatalytic performance.

**Fig. 12:**
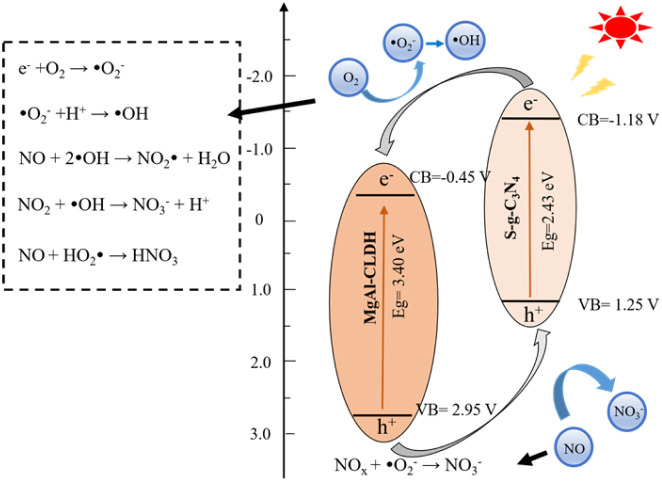
Photocatalytic NO_*x*_ degradation mechanism of S-g-C_3_N_4_/MgAl-CLDH.

To examine the electronic potentials of S-g-C_3_N_4_ and MgAl-CLDH, Mott–Schottky (M–S) tests were performed. The positive slope in the M-S plots indicates that both S-g-C_3_N_4_ and MgAl-CLDH have n-type semiconductor properties. According to the intersection point of slope and abscissa of the M–S plots, the flat band potentials (*vs.* SCE) for S-g-C_3_N_4_ and MgAl-CLDH are determined as −1.18 and −0.45 eV, respectively, which are similar to their conduction band (CB) potentials. By employing the formula *E*_g_ = *E*_VB_ − *E*_CB_, the valence band (VB) values (*vs.* SCE) for S-g-C_3_N_4_ and MgAl-CLDH were calculated as 1.25 and 2.95 eV, respectively. Then, a possible mechanism of photocatalytic NO_*x*_ degradation by the S-g-C_3_N_4_/MgAl-CLDH is proposed and illustrated in Fig. 12. Under light irradiation, electrons (e^−^) on the surface of S-g-C_3_N_4_ in the nanocomposite are excited and transiting from VB to CB. Furthermore, due to the higher CB position of S-g-C_3_N_4_ (−1.18 eV) compared to MgAl-CLDH (−0.45 eV), MgAl-CLDH acts as an efficient electron acceptor, capable of capturing electrons from the conduction band of S-g-C_3_N_4_. The photoexcited electrons are then transferred to the CB of MgAl-CLDH, accelerating electron separation and transfer, thereby enhancing the photocatalytic activity. On the surface of MgAl-CLDH, the generated electrons undergo a reduction reaction with absorbed O_2_, producing superoxide radicals (˙O_2_^−^). These radicals are then converted into hydroxyl radical (˙OH), which participates in catalytic oxidation reactions to degrade the target pollutant NO_*x*_. In addition, a portion of ˙O_2_^−^ can directly react with the NO_*x*_, thereby enhancing the reaction rate and promoting the separation of electrons and electron–holes (h^+^).

#### 3.2.3. Stability of S-g-C_3_N_4_/MgAl-CLDH

It has been found that elevated pH levels induce modifications in the structural characteristics of nanomaterials and surface properties, as well as the extent of ionization/dissociation pertaining to the molecules of adsorbed substances.^37,38^ The strong alkaline pore solution of cementitious materials may potentially affect the photocatalytic activity of photocatalyst. Herein, simulated concrete pore solution (SCPS) is used to evaluate the photocatalytic stability of S-g-C_3_N_4_/MgAl-CLDH.

**Fig. 13:**
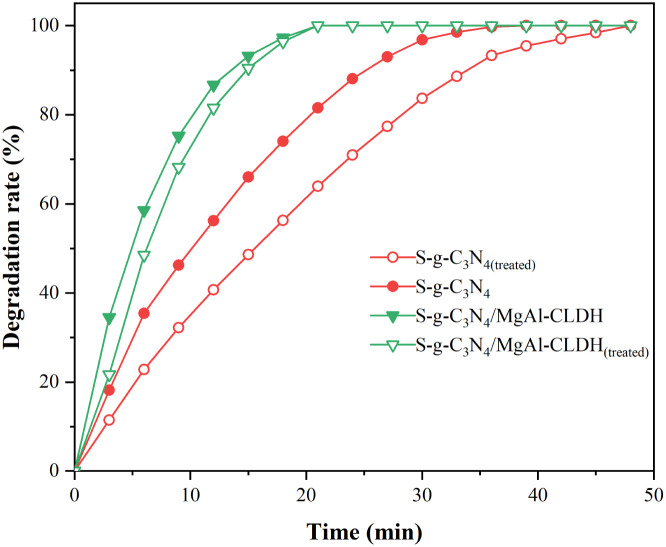
Photocatalytic degradation of NO_*x*_ by S-g-C_3_N_4_ and S-g-C_3_N_4_/MgAl-CLDH with or without SCPS treatment.

To this end, S-g-C_3_N_4_/MgAl-CLDH and S-g-C_3_N_4_ were immersed in two SCPS for a duration of 10 min. Subsequently, they were subjected to three cycles of rinsing with deionized water and vacuum-drying in an oven. The treated sample are denoted as S-g-C_3_N_4_/MgAl-CLDH_(treated)_ and S-g-C_3_N_4(treated)_. The degradation on NO_*x*_ of treated sample is shown in Fig. 13. After treatment, the degradation rate of S-g-C_3_N_4_ decreases significantly. At 12 min, the degradation rate of S-g-C_3_N_4_ is about 56.2%, and that of S-g-C_3_N_4(treated)_ is 40.7%, which decreases by 15.5%. In contrast, the degradation rate of S-g-C_3_N_4_/MgAl-CLDH is 86.7%, while that of the S-g-C_3_N_4_/MgAl-CLDH_(treated)_ after SCPS treatment is 81.5%, suggesting a gentle decrease by 5.2% only. The poor chemical stability of S-g-C_3_N_4_ in SCPS may be due to the adsorption of alkaline metal ions (K^+^, Na^+^, Ca^2+^) on the surface of the photocatalytic material in a strongly alkaline environment, leading to an increase in the rate of recombination of photogenerated electron–hole pairs and thus reducing the activity of the photocatalytic material.^39^ The direct contact between S-g-C_3_N_4_ and the alkaline solution was avoided by combining MgAl-CLDH. As a result, the metal cations were no longer available as compounding centers for the photogenerated electron–hole pairs, effectively mitigating significant loss in photocatalytic activity.

**Fig. 14:**
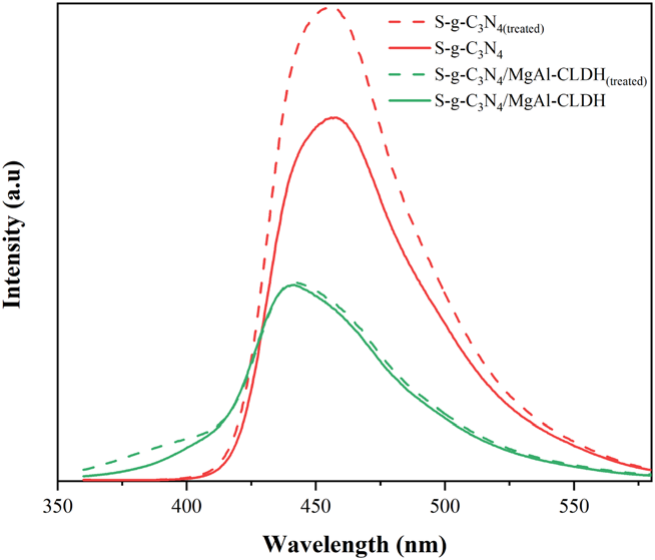
PL spectra of S-g-C_3_N_4_ and S-g-C_3_N_4_/MgAl-CLDH with or without SCPS treatment.

Photoluminescence (PL) spectra serve as an effective measure to characterize the photogenerated electron–hole pair recombination rate of the photocatalyst. Fig. 14 shows the PL spectra of S-g-C_3_N_4_ and S-g-C_3_N_4_/MgAl-CLDH with or without immersion in the SCPS. It is noticeable that S-g-C_3_N_4_/MgAl-CLDH has much lower PL intensity compared with S-g-C_3_N_4_, indicating lower photogenerated electron–hole pair recombination rate. In addition, the PL intensity of S-g-C_3_N_4_ is significantly enhanced after treatment, indicating that the S-g-C_3_N_4_ is susceptible to the strong alkaline environment. The photogenerated electron–hole pair recombination rate of S-g-C_3_N_4(treated)_ is increased and its photocatalytic activity is adversely affected. Similar result has been reported elsewhere.^40^ In contrast, the PL intensity of the S-g-C_3_N_4_/MgAl-CLDH material was only slightly enhanced after treatment, suggesting that S-g-C_3_N_4_/MgAl-CLDH has a high stability in SCPS. This can be attributed to the similarity of the structure of LDH with the hexagonal-layered hydration products of cement, including C_2_(A,F)H_8_, C_4_(A,F)H_13_ and C_4_(A,F)H_19_.^41^ Thus, MgAl-CLDH demonstrates excellent compatibility with cementitious materials and possesses favorable chemical stability in SCPS. Consequently, the introduction of MgAl-CLDH into S-g-C_3_N_4_ ensures that S-g-C_3_N_4_/MgAl-CLDH inherits respective advantages, weakening the adverse effects of highly alkaline environment on the photocatalytic activity of S-g-C_3_N_4_/MgAl-CLDH.

### 3.3. Photocatalytic activity in cement mortar

**Fig. 15:**
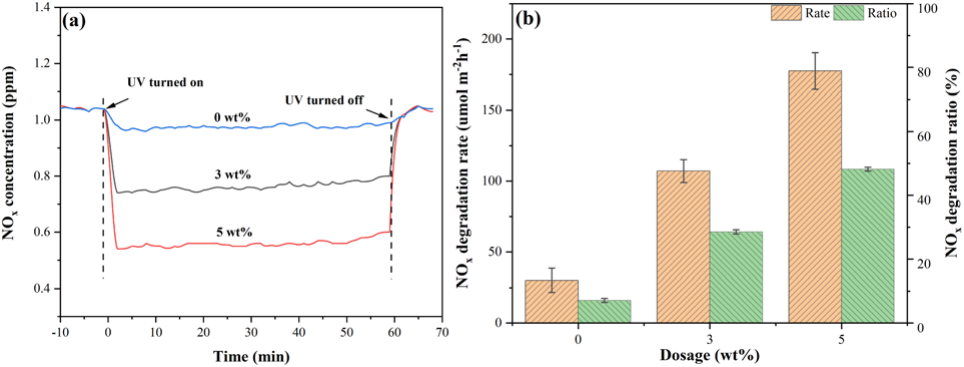
(a) NO_*x*_ degradation curves and (b) NO_*x*_ degradation rate and ratio of mortar internally mixed with different S-g-C_3_N_4_/MgAl-CLDH concentrations.

Fig. 15(a) shows the NO_*x*_ degradation curves of mortars internally mixed with S-g-C_3_N_4_/MgAl-CLDH. An instantaneous decrease in NO_*x*_ gas concentration at the onset of the photocatalytic reaction is observed. As the concentration of the photocatalyst increases, the decline in NO_*x*_ gas concentration becomes more pronounced. Notably, when the dosage of S-g-C_3_N_4_/MgAl-CLDH reaches 5 wt%, the instantaneous reduction in NO_*x*_ concentration is the highest.

Based on the data presented in Fig. 14(a), the NO_*x*_ degradations for cement mortar with different S-g-C_3_N_4_/MgAl-CLDH concentrations were calculated using eqn (3) and (4) given in Subsection 2.5.2. The results are shown in Fig. 15(b). To be specific, the NO_*x*_ degradation rates for mortars containing 0 wt%, 3 wt% and 5 wt% of S-g-C_3_N_4_/MgAl-CLDH are 30.1, 107.1 and 177.5 μmol m^−2^ h^−1^, respectively. Similarly, the NO_*x*_ degradation ratios for the corresponding mortars are 7.2%, 28.53% and 48.2%, respectively. This indicates that the increase in the dosage of S-g-C_3_N_4_/MgAl-CLDH results in an increase in the NO_*x*_ degradation rate of photocatalytic cement mortar. The observed phenomenon can be attributed to the constrained accessibility of active sites of the photocatalyst embedded in the cementitious matrix at lower concentrations of S-g-C_3_N_4_/MgAl-CLDH. Nevertheless, with the increase of S-g-C_3_N_4_/MgAl-CLDH content, the population of active sites on the surface of the cementitious material increases, thus progressively enhancing its ability to degrade NO_*x*_ through photocatalysis. Notably, photocatalytic mortar with 5 wt% S-g-C_3_N_4_/MgAl-CLDH exhibits the highest NO_*x*_ degradation rate and ratio.

**Fig. 16:**
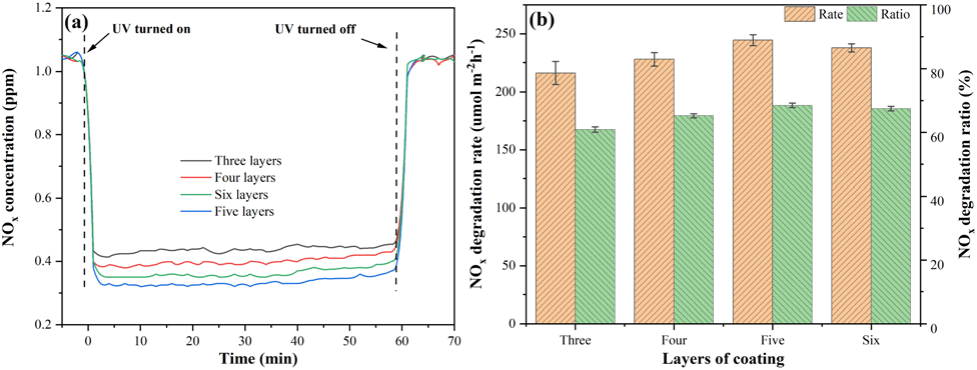
(a) NO_*x*_ degradation curves and (b) NO_*x*_ degradation rate and ratio of mortar coated with S-g-C_3_N_4_/MgAl-CLDH under different coating layers.

Fig. 16(a) shows the NO_*x*_ degradation curves of mortars coated with S-g-C_3_N_4_/MgAl-CLDH. With the increase of coating layers, the magnitude of instantaneous NO_*x*_ concentration reduction shows an initial increase followed by a decrease. To be specific, at 5 layers of coating, the greatest reduction in NO_*x*_ concentration is achieved. Fig. 16(b) shows the NO_*x*_ degradation rate and degradation ratio of photocatalytic cement mortar under different coating layers. It is obvious that the photocatalytic cement mortar coated with 5 layers of S-g-C_3_N_4_/MgAl-CLDH exhibits the highest NO_*x*_ degradation rate of 244.41 μmol m^−2^ h^−1^ and a degradation ratio of 68.5%. In comparison, the mortar with 3 layers of S-g-C_3_N_4_/MgAl-CLDH shows the lowest NO_*x*_ degradation rate and degradation ratio, as 216.29 μmol m^−2^ h^−1^ and 61.5%, respectively.

The reason for the aforementioned phenomenon is described as follows. When the surface of the cement mortar is coated with a small quantity of photocatalytic material, the light absorption characteristics of the S-g-C_3_N_4_/MgAl-CLDH coating layer are weak, leading to fewer absorbed photons and fewer generated reactive oxygen species (ROS), resulting in low photocatalytic activity. For higher coating amount of S-g-C_3_N_4_/MgAl-CLDH on the cementitious substrate surface, the light absorption characteristics of the coating layer is enhanced, resulting in an increased generation of ROS and an improvement in photocatalytic activity. Additionally, the thickness of the S-g-C_3_N_4_/MgAl-CLDH coating layer is also a crucial factor controlling the electron transfer and light absorption efficiency. When the coating reaches a certain thickness, the light absorption efficiency of the photocatalytic cementitious material saturates, and the photocatalytic activity reaches a plateau. In addition, multiple coatings on the cementitious surface gradually reduce the porosity, leading to a decline in its adsorption capacity. The degradation of NO_*x*_ in the cementitious material is a synergistic effect of its adsorption and degradation. A decrease in adsorption performance results in a reduction of internal NO_*x*_ concentration. Therefore, after 5 layers of coating, the photocatalytic capacity for NO_*x*_ degradation in the coated mortar gradually declines with further increase of coating.

**Fig. 17:**
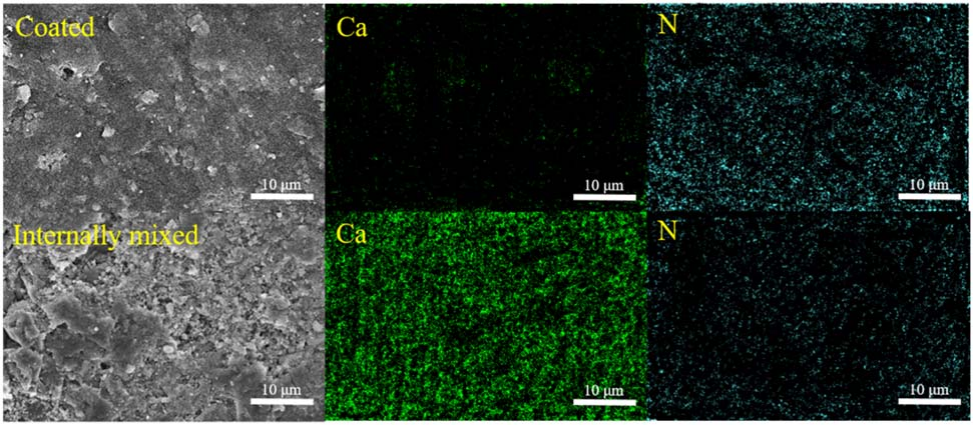
SEM images and elemental mapping analyses of the mortar coated or internally mixed with S-g-C_3_N_4_/MgAl-CLDH.

The effects of the internal mixing and coating method on the degradation of NO_*x*_ in cement mortar were further elucidated by investigating the distribution of S-g-C_3_N_4_/MgAl-CLDH on the surface of the cement mortar. Fig. 17 shows the SEM images of mortar coated with 5 layers of S-g-C_3_N_4_/MgAl-CLDH and internally mixed with 5 wt% S-g-C_3_N_4_/MgAl-CLDH. The surface of the mortar coated with S-g-C_3_N_4_/MgAl-CLDH is dominated by nitrogen (N), which is rich in S-g-C_3_N_4_/MgAl-CLDH, while the concentration of calcium (Ca) is very low. This indicates that S-g-C_3_N_4_/MgAl-CLDH is evenly scattered on the surface of cement mortar and the loading amount is abundant. The increased exposure of S-g-C_3_N_4_/MgAl-CLDH on the surface of the cement mortar provides more opportunities for contact with NO_*x*_, thereby facilitating the photocatalytic oxidation reaction. Whereas, the surface of mortar internally mixed with S-g-C_3_N_4_/MgAl-CLDH is rich in Ca and poor in N, suggesting insufficient loading amount. It is concluded that in terms of photocatalytic performance, the coating method is more efficient, resulting in higher NO_*x*_ degradation.

## 4. Conclusions

A novel photocatalytic nanocomposite S-g-C_3_N_4_/MgAl-CLDH was prepared using electrostatic self-assembly method. The application potential of S-g-C_3_N_4_/MgAl-CLDH in cement-based materials was evaluated based on measurements of physicochemical properties, photocatalytic performances and chemical stability of S-g-C_3_N_4_/MgAl-CLDH after immersing in simulated concrete pore solution (SCPS). Besides, photocatalytic performances of cement mortars loaded with S-g-C_3_N_4_/MgAl-CLDH were investigated. The main conclusions can be drawn as follows:

(1) A significant change in the lattice spacing between S-g-C_3_N_4_ and MgAl-CLDH was revealed by TEM technique and the formation of a heterogeneous structure between the two materials was confirmed. The successful synthesis of S-g-C_3_N_4_/MgAl-CLDH with high degree of crystallinity was demonstrated by XRD and FTIR.

(2) Compared to S-g-C_3_N_4_, the S-g-C_3_N_4_/MgAl-CLDH exhibited a narrower bandgap (2.45 eV), a lower rate of photogenerated electron–hole pairs recombination and a 1.94-fold increase in specific surface area. After 30 min of visible light irradiation, the degradation rate of RhB by S-g-C_3_N_4_ reached 88% only, whereas S-g-C_3_N_4_/MgAl-CLDH achieved a complete degradation of NO_*x*_. After 21 min of visible light irradiation, the NO_*x*_ degradation rate of S-g-C_3_N_4_/MgAl-CLDH achieved 100% while that of S-g-C_3_N_4_ was merely 81.5%.

(3) After being submerged in SCPS, the photocatalytic NO_*x*_ degradation efficiency of S-g-C_3_N_4_ was decreased by 15.5% after 12 min of illumination, whereas S-g-C_3_N_4_/MgAl-CLDH exhibited only a slight decrease of 5% in degradation efficiency. The superior photocatalytic stability of S-g-C_3_N_4_/MgAl-CLDH is verified, which can be attributed to the excellent compatibility and stability of MgAl-CLDH in SCPS. Meanwhile, MgAl-CLDH serves as a barrier avoiding the direct contact of S-g-C_3_N_4_ and alkaline substances.

(4) The photocatalytic performance of the internally mixed mortar in degrading NO_*x*_ improves gradually with an increase in the dosage of S-g-C_3_N_4_/MgAl-CLDH. The mortar with 5 wt% of S-g-C_3_N_4_/MgAl-CLDH exhibits the highest NO_*x*_ degradation rate, reaching 48.2%. The NO_*x*_ degradation performance of mortar improves as the coating increases up to 5 layers, but a decline in NO_*x*_ degradation rate after 5 layers of coating is found owing to the reduced porosity of mortar because of excessive coating.

(5) In terms of photocatalytic performance, the coating method is more efficient, enabling higher NO_*x*_ degradation, than the internal mixing method.

## Author contributions

Conceptualization, Z. Y., X. X. and Y. Z.; data curation, Z. Y., X. X. and S. L.; formal analysis, Z. Y. and X. X.; investigation, X. X. and S. L.; methodology, Z. Y. and X. X.; project administration, Y. Z., B. B. and G. C. M.; supervision, Y. Z., B. B. and G. C. M.; validation, Y. Z. and S. L.; writing-original draft, Z. Y. and X. X.; writing-review and editing, Y. Z. All authors have read and agreed to the published version of the manuscript.

## Conflicts of interest

The authors declare that they have no known competing financial interests or personal relationships that could have appeared to influence the work reported in this paper.

## Supplementary Material
